# Saunders’ Question Compends

**Published:** 1890-12

**Authors:** 


SAUNDERS' QUESTION COMPENDS, Nos. 8 and 9. Essentials of Practice of Medicine; arranged in the form of Questions and Answers. Prepared especially for Students of Medicine. By HENRY MORRIS, M. D., late Demonstrator Jefferson Medical College, Philadelphia; Visiting Physician to St. Joseph's Hospital, etc., etc. With a very complete Appendix on the Examination of Urine. By Lawrence Wolff, M. D., Demonstrator of Chemistry, Jefferson Medical College. Philadelphia: W. B. Saunders. 1890.

This little book is one of a kind that should never be published. The various Quiz Compends, Question Compends, and Essentials of the various departments of medical knowledge that have appeared in the last few years, have done more than any one thing to make medical education superficial. The students, instead of devoting themselves to the study of the principles of the practice of medicine, and the application of these principles to individual cases, try to learn by note, by means of question and answer, the diagnosis and therapy of each disease as a separate entity; and these kind of books aid them in acquiring just such superficial knowledge. We quote from the preface:

This little volume is intended as an *aid* to the advanced student of medicine who is preparing for his degree, or to the young practitioner in diagnosing affections, or selecting the remedy for them. The author hopes it will be distinctly understood by the student that this book is not intended to, nor can it, replace the larger text-books in general use.

As a matter of fact, whether the author intends it or not, such little books do take the places of the larger text-books, and a great many students "cram" for examinations and succeed in passing by the aid of just such books; students who, without such aid, would not succeed in passing at all, or, at any rate, would take more years of careful study to accomplish their object, and, in consequence, would be much better physicians and broader-minded men of greater culture, and, therefore, more useful citizens than is possible for them to become under the "cram-for-a-degree" kind of education that is fostered by Question Compends and their congeners.

For this sort of book the one before us answers its purpose very well. The appendix on The Chemical and Microscopic Examination of the Urine for Clinical Purposes, which is excellent, makes it of more value than most of its kind.

The binding and letter-press are very good, but the illustrations are not very clear, the wood-cut type apparently being old, or not well done. DeL. R.

